# Hyper-Angle Exploitative Searching for Enabling Multi-Objective Optimization of Fog Computing

**DOI:** 10.3390/s21020558

**Published:** 2021-01-14

**Authors:** Taj-Aldeen Naser Abdali, Rosilah Hassan, Azana Hafizah Mohd Aman, Quang Ngoc Nguyen, Ahmed Salih Al-Khaleefa

**Affiliations:** 1 Centre for Cyber Security, Faculty of Information Science and Technology (FTSM), Universiti Kebangsaan Malaysia, UKM, Bangi 43600, Malaysia; P94546@siswa.ukm.edu.my (T.-A.N.A.); azana@ukm.edu.my (A.H.M.A.); ahmed.salih89@siswa.ukm.edu.my (A.S.A.-K.); 2 Department of Communications and Computer Engineering, Faculty of Science and Engineering, Waseda University, Tokyo 169-8050, Japan; quang.nguyen@aoni.waseda.jp

**Keywords:** fog computing, task allocation, multi-objective optimization, evolutionary genetics, hyper-angle, crowding distance

## Abstract

Fog computing is an emerging technology. It has the potential of enabling various wireless networks to offer computational services based on certain requirements given by the user. Typically, the users give their computing tasks to the network manager that has the responsibility of allocating needed fog nodes optimally for conducting the computation effectively. The optimal allocation of nodes with respect to various metrics is essential for fast execution and stable, energy-efficient, balanced, and cost-effective allocation. This article aims to optimize multiple objectives using fog computing by developing multi-objective optimization with high exploitive searching. The developed algorithm is an evolutionary genetic type designated as Hyper Angle Exploitative Searching (HAES). It uses hyper angle along with crowding distance for prioritizing solutions within the same rank and selecting the highest priority solutions. The approach was evaluated on multi-objective mathematical problems and its superiority was revealed by comparing its performance with benchmark approaches. A framework of multi-criteria optimization for fog computing was proposed, the Fog Computing Closed Loop Model (FCCL). Results have shown that HAES outperforms other relevant benchmarks in terms of non-domination and optimality metrics with over 70% confidence of the t-test for rejecting the null-hypothesis of non-superiority in terms of the domination metric set coverage.

## 1. Introduction

Internet of Things (IoT) has been used in several fields such as health care, environmental engineering, transportation, and safety [1,2]. The idea behind IoT is to connect physical items to the virtual world, so they can be controlled remotely and act as physical access points to Internet services [3]. These devices increased rapidly around the world and generate a huge amount of data, termed Big Data (BD) [4,5]. One of the fundamental challenges in IoT is the data transmissions [6,7] to the Cloud Computing (CC), which indicate to the infrastructure where both data storage and processing operate outside of the IoT devices [8,9].

**Figure 1:**
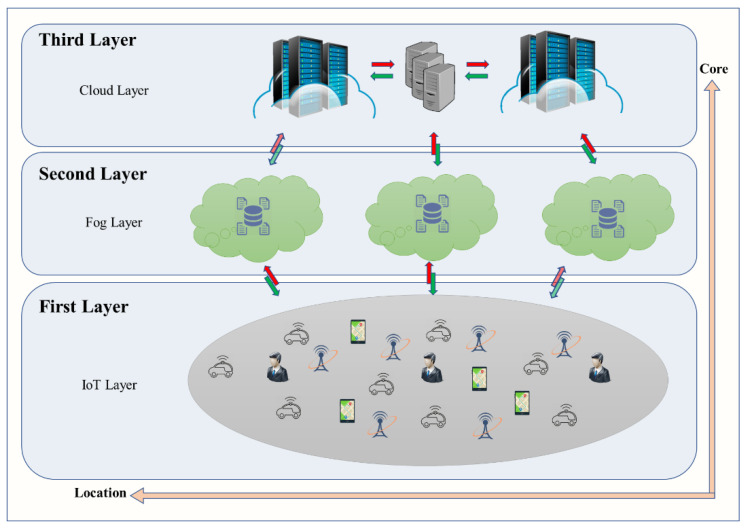
A basic conceptual framework of IoT, cloud computing, and fog computing.

CC data center is far from end-user, then causes high latency and affects the actual time constraints in many applications [10]. Therefore, CISCO [11] suggests the new paradigm Fog Computing (FC) to ensure reliable sending and receiving data between the Cloud and IoT devices [12]. Figure 1 gives a conceptual elaboration of the architecture of IoT, CC, and FC. The first layer is the IoT environment, this layer close to the user and the physical environment. It contains several devices such as mobile phones, sensors, smart cards, readers, and smart vehicles. The second layer fog layer this layer is located on the edge of the network means between IoT and cloud computing. This layer contains a huge number of fog nodes which generally including routers, gateways, switches, access points, base stations, and specific fog servers. The third layer is the cloud computing layer and consists of several effective servers and storage devices and provides various application services for smart homes, smart transportation, smart factories, and so on.

The distributed nature of FC and the relatively limited computation, energy, and communication power of its nodes have motivated researchers to assure its load balancing aspect when various applications are required to be executed in FC. The load balancing of fog computing is accomplished by a set of methodological approaches named Task Allocation (TA) [13] in the literature. The term TA indicates allocating various network nodes optimally to execute a given task or application while maintaining various objectives. In the context of TA for FC, we are interested in dividing the given task into a set of sub-tasks with the independency aspect and dividing them on the network nodes with matching various constraints. Next, they will be presented with a mathematical model for calculating the various fog measures, including energy efficiency, cost-effectiveness, time latency, stability, and reliability. Having the ability to evaluate the candidate solution from the optimization and to provide its objectives values, we call Fog Computing Closed Loop (FCCL). This type of problem is regarded as a Non-Deterministic Polynomial Hard Problem (NP-hard) [14], which makes it a challenging optimization problem. This is due to the huge number of combinations of nodes’ task allocation and the various conditions of the nodes and the tasks. Typical approaches for solving such a problem use a meta-heuristic family of optimization algorithms, and more specifically, the multi-objective type of meta-heuristic was enhanced to apply for fog computing to hold the huge number of tasks and set them based on their priority.

Multi-Objective Optimization (MOO) algorithms [15,16] aim at optimizing many objectives’ functions using heuristic random searching in order to find a set of non-dominated solutions [17]. There is a high similarity between single objective [18] and multi-objective meta-heuristics [19,20] in the aspect of relying on a random pool of generated solutions, evaluating them, and selecting the best among them to generate off-spring. However, the essential difference between the single objective and multi-objective heuristic searching is the means of evaluating solutions. More specifically, in the multi-objective searching, the solutions are evaluated based on ranks that include a sub-set of non-dominated solutions instead of simple fitness value as in the single-objective optimization. Consequently, the goal of the MOO algorithm is to explore the solution space for finding maximum coverage of non-dominated solutions.

The goal of this article is to develop an optimization framework for computational fog computing. We aim to enable non-dominated optimization for fog computing by assuring high domination of the resulted decisions in terms of various performance metrics, which gives the decision-maker more flexibility as well as high achieved performance. Specifically, the integration of a novel hyper-angle exploitive searching optimization with the crowding distance of Non-Dominated Sorting Genetic Algorithm II (NSGA-II) in the context of fog computing optimization assists in providing more dominant solutions in terms of the fog measures that the decision-maker aims at optimizing. The article presents the following contributions.

- Proposing a fog computing optimization framework with multi-criteria perspectives. The multi-criteria cover the following metrics: Time Latency, Energy Consumption, Energy Distribution, Renting Cost, and Stability.
- Developing a novel optimization algorithm based on meta-heuristic genetic. The developed algorithm supports exploitive searching based on the hyper-angle indicator. We designate it as Hyper-Angle Exploitive Searching (HAES).
- Formulating a novel Fog Computing Closed Loop (FCCL) mathematical function and using HAES for optimizing it after discretization.
- Designing an adaptive objective partitioning by activating the sub-set of objectives at each iteration out of the entire objectives.
- Evaluating the developed HAES based on multi-objective optimization performance metrics and benchmarking mathematical functions and evaluating the optimized FCCL based on HAES, then analyzing its performance in comparison with other relevant optimization benchmarking algorithms.

## 2. Background and Literature Review

The article is focusing on multi-objective optimization for FC. Hence, the literature contains two phases. Firstly, the related work of the MOO algorithms is presented in Section 2.1, and Section 2.2 provides the related works of MOO fog computing optimization in.

### 2.1. MOO Algorithms

The studies on meta-heuristic-based MOO in the literature contain various approaches. Different criteria and techniques are used to generate the dominant Pareto Front (PF) and provide extensive exploration. In [21], a fitting function or interpolation method was applied from a finite set of objective values to calculate PF by selecting the individuals that have the shortest distance to the reference points based on the error matrix. The two algorithms, called MOGA/fitting and MOGA/interpolation, dealt with MOO without focusing on attaining the optimal solutions. Bao et al. [22] proposed Hierarchical NDS (HNDS), which focuses on reducing the number of comparisons in the search. HNDS initially sorts all the candidate solutions in ascending order, depending on their first objective. Next, HNDS compares the first solution with the rest of the candidate solutions, one by one, to make a speedy distinction by realizing different superiority solutions and then avoid the high number of unnecessary comparisons.

Other notable studies have extended the existing single-objective searching algorithms to multi-objective ones by introducing the concept of NSGA-II, which is fast NDS with crowding distance. This extension applies to Multi-Objective Vortex Searching (MOVS), which was proposed in [23]. MOVS uses the inverse incomplete gamma function with a parameter ranging from 0 to 1 to spread solutions over the PF. improved NSGA-II to make it more efficient and have better diversity by presenting a more efficient implementation of NDS, namely the dominance degree approach for NDS. Part and Select Algorithm (PSA) was also proposed to maintain diversity, and the entire algorithm after being integrated into NSGA-II was called Diversity DNSGA2–PSA. Additionally, several researchers have added a local search strategy to NSGA-II [24]. For example, the study in [25] proposed Heavy Perturbation (HP)-based NSGA-II. Two objectives, the size and total weight of a clique, were considered. In particular, the larger the size of a clique in terms of set inclusion is and the higher the total weight is, the better a solution is. HP-NSGA-II is then dedicated to the clique problem of a weighted graph with weights of vertices in which the perturbation is conducted by either improving a selected elite with a local search procedure or swapping its left and right parts.

Several types of research work also developed nature-inspired models for MOO. For instance, an improved method of GA based on an evolutionary computational model, namely the Physarum-Inspired Computational Model (PCM), was proposed in [26]. The initialization of the population used prior knowledge of PCM. Hill climbing was also used to improve the diversity of solutions, and the traveling salesman problem, which is one of the most classical NP-hard problems in combinatorial optimization, was utilized. Apart from improving the optimization of found solutions, several researchers have aimed at improving the searching speed. In the same context, [27] proposed an algorithm for MOO and compared it with four other competing algorithms on three different datasets to reduce the optimization complexity for a large number of objectives from $O\left(N{\;\log}^{M-1}N\right)$ to $O\left(MN\;\log\;N+MN^{2}\right)$, where M denotes the number of objectives and N denotes the number of solutions. The algorithm removes unnecessary comparisons among solutions to improve the running time.

The work in [28] added the angle concept to crowd distance searching to balance the searching procedure among all angles. Other researchers have also used the framework of NSGA-II with different extensions. For example, [29] used a set of reference points while searching to maintain diversity. Then, from previous approaches, the concept of crowd distance, when combined with angle searching, achieves the extensive scope of the search. Specifically, authors in [30] have used range angle as a criterion to balance the search, then using it in finding criterion solutions as the goal of the study.

Overall, the previous research works that have focused on meta-heuristic for multi-objective aimed at incorporating various criteria for accomplishing exploration as well as exploitation. The crowding distance of NSGA-II is effective for exploration, while the angle searching was used in MOGA-AQCD as an additional base for the crowd-distance exploration. However, the angle usage for exploitation has not been explicitly considered and performed by the existing studies. This article then aims at tackling this aspect by proposing a novel MOO searching that incorporates angle searching for exploitation.

Particularly, the present paper proposes a MOO searching algorithm that uses crowding distance for exploration and angle searching for exploitation. The proposal optimizes the exploitation by selecting solutions from angular sectors that have the maximum found solutions. The crowding distance is also used for exploration; however, we aim at avoiding redundant operators for exploration. This goal is achieved by considering angle searching for exploitation, provided that the crowding distance has successfully played its role in the exploration process. To our knowledge, this is the first meta-heuristic searching algorithm for MOO that jointly considers and optimizes the angle criterion for exploitation and crowding distance for exploration at the same time. In the next section, we present the system models and the research background.

### 2.2. Fog Computing Optimization

Solving IoT challenges of data processing within real-time constraints have created the need to not rely on cloud network for processing. As a result, the concept of Fog Computing was first introduced by Cisco in 2012. However, congested networks, high latency in service delivery, and poor Quality of Service (QoS), non-stability, and increased cost have been experienced [31]. Such challenges have motivated researchers to focus on fog computing optimization.

The literature contains a significant amount of algorithmic works for fog computing optimization. Each work has focused on certain aspects of the fog network and followed a certain approach for optimization. While some work has tried to include more practical aspects of fog computing needs and nature, others were more simplified and ignored some crucial matters. In the work of [32], the authors have represented the fog computing optimization as a scheduling problem, where the algorithm has to assign tasks to nodes with assuring two objectives the stability and speed. Their model ignores energy and cost matters, which are considered to be crucial aspects of fog computing. On the other side, they used classical multi-objective optimization NSGA-II to solve their model without significant changes to explore the solution space more efficiently and find more dominant solutions. We find that other models have considered energy and cost like the work of [33]; however, there is no consideration of stability or reliability for finishing the work. Similarly, the work of [34] has included energy and latency while ignoring cost and reliability, while the work of [35] has included time latency and cost as objectives and it ignored energy and reliability.

A summary of the covered objectives of each model is given in Table 1. To the best of our knowledge, there is no developed model for fog computing optimization including four objectives: time latency, energy, cost, and reliability at the same time. Such inclusion implies more challenging multi-objective optimization. On the other side, all the previous works have applied NSGA-II and other similar non-dominated searching optimization without development in the searching aspect, which is needed because of the non-convex nature of the problem and a huge number of constraints resulting in the optimization surface non-linear and non-convex with NP-hard nature.

## 3. Proposed Methodology

This section presents the developed method for accomplishing the goal of the article. It starts with presenting the problem formulation of optimization and fog computing framework was provided in Section 3.1. Next, in Section 3.2, we provide the algorithm named hyper-angle exploitive searching. The fog computing closed-loop model is given in Section 3.3. Table 2 elaborates on the mathematical terms used in the article.

### 3.1. Problem Formulation of Optimization and Fog Framework

Assume that we have a tuple $x=\left(x_{1},x_{2},\ldots x_{n}\right)\in X,$ where $X\subseteq R^{n}$ and a tuple $y=\left(y_{1},y_{2},\ldots y_{m}\right)\in Y$ where $Y\subseteq R^{m}$ in which the following constraints are held:

(1) $$y_{1}=f_{1}\left(x_{1},x_{2},\ldots x_{n}\right)$$

(2) $$y_{2}=f_{2}\left(x_{1},x_{2},\ldots x_{n}\right)$$

(3) $$y_{m}=f_{m}\left(x_{1},x_{2},\ldots x_{n}\right)$$

In such a scenario: x is called the decision vectors; y is the objective vector. X is the solution space, and Y is the objective space to model a minimization problem, with two vectors a and b. We call b dominates a, denoted as a≺b iff:

(4) $$\{\begin{array}{c} \forall \;i\;\in \left\{1,2,\ldots m\right\}:f_{i}\left(a\right)\leq f_{i}\left(b\right)\\ \exists \;j\in \left\{1,2,\ldots m\right\}:f_{j}\left(a\right)<f_{j}\left(b\right) \end{array}$$

The domination of b over a is applied when b is superior over a with at least one of the objectives j, and b is not worse than a in the remaining objectives i.

### 3.2. Hyper-Angle Exploitive Searching HAES

This section presents a hyper angle exploitive searching HAES algorithm. Firstly, we present its working principle and the difference between HAES and MOGA-AQCD [30] in Section 3.2.1. Secondly, we present the objective partitioning in Section 3.2.2. Lastly, the algorithm of HAES in Section 3.2.3.

#### 3.2.1. Working Principle and the Difference between HAES and MOGA-AQCD

Both the proposed HAES and MOGA-AQCD use the concept of angle quantization for searching, which is based on dividing the space into equal-angle sectors and building a histogram that calculates the number of solutions selected for each sector. However, HAES behaves differently from MOGA-AQCD in terms of the selection of the new solutions. MOGA-AQCD favors solutions located in the least angular sector in terms of the previously selected solutions when two solutions are non-dominated with each other. In contrast, HAES favors solutions located in the maximum angular sector in terms of the previously selected solutions. Typically, the MOGA-AQCD concept is to perform extensive exploration to yield substantial optimal solutions, whereas the HAES concept is that sectors that cover suitable solutions in the past are also likely to be rich in the future. We then provide an example to explain the critical difference between HAES and MOGA-AQCD regarding the searching concept.

**Figure 2:**
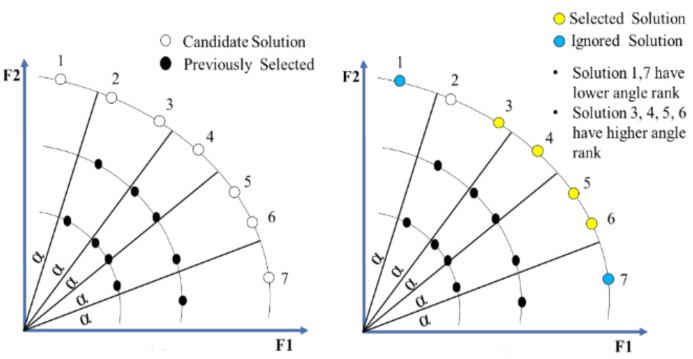
The selected solution in solution space by HAES.

The concept of HAES is depicted in Figure 2. The solution space is decomposed into a set of angular sectors. Each angular sector contains a set of solutions. The already found solutions are marked with black bullets and the candidate solutions are represented with white bullets. HAES selects the solutions that are located in the highest angular sector with respect to the number of solutions. We mark the selected solutions with yellow bullets and the ignored solutions with blue bullets.

#### 3.2.2. Objectives Partitioning

The multi-objective optimization when working on a high number of objectives requires searching within a wide objective space, which makes it challenging to converge toward the boundary of the objective space. Hence, we do boundary searching mechanisms by activating the sub-set of objectives at each iteration out of the entire objectives. We name it objective partitioning; its role is to reach the boundary of the solution space with respect to the activated objectives. We select at each iteration of the optimization size k<m, where m denotes the number of objectives, and we use it for evaluating the solutions, sorting them, and selecting non-dominated ones. The sub-set of objectives is selected randomly at each iteration using a uniform distribution.

#### 3.2.3. Algorithm of HAES

The general algorithm of HAES is presented in Algorithm 1. The algorithm takes the number of generations NGen, the number of solutions NSol, the sector range value SectorRange, and the set of objectives SoB as inputs, the size of objectives partitioning. The output of the algorithm is the Pareto front ParetoFront. As can be seen in Algorithm 1, the algorithm starts with the initialization of the first population in line 10, keeping it as a previous population in line 11, initialization of the counter of the population in line 12, initialization of the angle range rank in line 13, and initialization of crowding distance in line 14. Next, an iterative while loop is performed until the number of generations is finished. The loop is composed of calling for the evaluation of the solutions in the previous generation using the objective partitioning in function selectSubSet (line 15) and the objective function calculation in the function evaluate (line 16), updating the crowding distance using the function updateCrowdingDistance (line 17), updating the ranges using the function updateRanges (line 18), selecting the elites that are responsible for generating the off-spring using selectElites (line 20), generating the off-spring using the function geneticOperations (line 22), and the concatenation of the parents and their off-spring using the concatenation operator || (line 23), and finally the new population is selected again from the resulted concatenated using the electElites one more time (line 25). This process is repeated until the total iterations are finished, then the Pareto front of the last generated solution is the result of the algorithm, as presented in line 26.
**Algorithm 1** Pseudocode of the HAES Algorithm1.**Input:**2.  $N_{Gen}$                  //Number of Generations 3.    $N_{Sol}$                 //Number of Solutions 4.  *SectorRange*               //Sector Range5.  SoB = *f_i_,* where *i* = 1, 2, …, *m*;      //Set of Objectives 6.  *K*                    //size of objectives partitioning 7.**Output:**8.  *ParetoFront*            //Found Pareto Front9.**Start:**10.   $P_{0}$ = InitiateFirstPopulation$\;N_{Sol}$;      //generate first population randomly11.   populationPrevious = $P_{0}$;      //first population is the previous population12.   counterOfGeneration = 1;13.   angleRangeRank = zeros (1, 2*π*/*SectorRange*) //initialize the angle range rank14.   **while** (CounterofGeneration < $N_{Gen}$)15.    
SSoB
**=**
selectSubSet(SoB,k)
16.    [solutionsRanks,objectiveValues] = evaluate (populationPrevious,SSoB)17.    [*crowdingDistance*] = updateCrowdingDistance (populationPrevious,objectiveValues)18.    [*angleRangeRank*] = updateRanges (populationPrevious,solutionsRanks,19.    *SectorRange,angleRangeRank*, SoB) //select $N_{Sol}$ from the previous solutions20.    selected Elites = *selectElites*
21.    ($P_{0}$,solutionsRanks,*angleRangeRank,crowdingDistance*, $N_{Sol}$)22.    offSpring = geneticOperations (selected Elites)23.    combinedPop = *selectedElites* || offSpring sortedCombinedPop = 24.  NonNominatedSorting (combinedPop)25.  $P_{New}$ = selectElites (sortedCombinedPop, *angleRangeRank*,NSol)26.  populationPrevious = $P_{New};$
27.  CounterofGeneration++;28.  **end while**29.**End**

The algorithm calls three essential functions: updateCrowdingDistance(), updateRanges(), and selectElites(). We provide the details of each of them in Algorithm 2, Algorithm 3, and Algorithm 4, respectively. For the updateCrowdingDistance(), the algorithm (detailed in Algorithm 2) takes the number of solutions NSolutions and the objective values objectiveValues as input, and provides the set of crowding distance crowdingDistance. The algorithm starts with the initialization of the set of the crowding distance with the size of solutions NSolutions (line 7). Next, the two extreme solutions are assigned the value of infinity (line 8). Afterward, the algorithm sorts the solutions as the separated lists according to their objective values (line 9). Then, the algorithm updates the crowding distance in an accumulated way, corresponding to the difference between each objective of a solution and the value of its next solution in the sorted list (line 11).
**Algorithim 2** Pseudocode of calculating the crowding distance1.**Input:**2.  
$N_{Sol}$
3.  *objectiveValues*
4.**Output:**5.   *CrowdingDistance*6.**Start:**7.   *crowdingDistance* = zeros ($N_{Sol}$);8.   *crowdingDistance* (1) = *crowdingDistance* ($N_{Sol}$) = ∞9.   **for** (each i objective of *objectiveValues)* sortedSolutions = sort ($N_{Sol}$,i);10.    **for** (solution j from 2 to $N_{Sol}$) 11.      *crowdingDistance* (j) = *crowdingDistance*(j) + *objectiveValues*(i) − *objectiveValues*(i − 1);12.    **end for**13.   
**end for**
14.**End**

The updateRanges() function is provided in Algorithm 3. It takes three variables: Solutions, SectorRange, and *SoB,* as input. Additionally, it gives angleRangeRank as output. The approach of obtaining angleRangeRank is based on performing an iterated loop in the input Solutions and updating the counter of each sector in the SectorRange that contains the solution, as presented in the for loop from line 10 to line 13.
**Algorithim 3** Pseudocode of updating the angle range rank1.**Input:**2.  *Solutions*
3.  *SectorRange*
4.  *SoB*5.**Output:**6.  
angleRangeRank
7.**Start**8.    L = length (Solutions)9.     *angleRangeRank* = zeros (360/*SectorRange*)10.    **for** (i = 1 to *L*)11.      $A_{i}\;$= angle (solution(i))//angle of solution i12.      **angleRangeRank** (j) = map ($A_{i}$, *SectorRange*) + *angleRangeRank* (j)13.    **end for**
14.    return angleRangeRank 
15.**End**

The final procedure receives the pool of solutions Pool of Solutions, the rank of solution Rank, the angle range rank AngleRangeRank, the array of the crowding distance CrowdingDistance, and the number of solutions to be selected N as input and provides selected solutions (Algorithm 4). The procedure performs an iterated loop for N times, where it selects two solutions in each time and calculates three measures for each solution: rank, angle range rank, and crowding distance. Next, the selection function determines which one has a better rank (line 17), better angle range rank (line 19), and better crowding distance (line 21). Then, the selection process is applied by checking the condition (line 22–24) to identify which favors a solution that has a better rank. In the case that two solutions have the same rank, then the solution with better angle range rank is selected. If the two solutions both have the same values of rank and angle range rank, then the approach will select the solution that has better crowding distance. In addition, the definition of “better” is provided for rank in line 17, for angle range rank in line 19, and for crowding distance in line 21. The detail of the algorithm for selecting the elites is shown in Algorithm 4.
**Algorithim 4** Pseudocode of selecting the elites1.**Input:**2.  *Pool of Solutions*
3.  *Rank*
4.  *AngleRangeRank*5  CrowdingDistance
6.  N                    //number of the selected solutions7.**Output**:8.  *selected solutions*
9.**Start:**10. **for** (solution = 1 to N)            //number of the selected solutions 11.  Select two individuals *A*, *B* randomly for an individual12.  Compute Non-domination rank (*rank*)13.  Compute Crowding distance (*distance*)14.  Compute Angle rank level (*angle Range Rank*)15.
16.      //**Compare Solutions**
17.  betterRank = *A*_*rank* < *B*_*rank*18.  sameRank = *A*_*rank* == *B*_*rank*19.  better*AngleRangeRank* = *A_angleRangeRank* > *B_angleRangeRank*20.  sameAngleRangeRank = *A_angleRangeRank* == *B*_*angleRangeRank*21.  better*CrowdingDiandstance* = *A*_distance > *B*_distance22.  ***if*** (betterRank) 23.   *or* (sameRank and betterAngleRangeRank)24.   *or* (sameRank and sameAngleRangeRank and betterCrowdingDistance)25.  **then**26.      add *A* to the selected solutions27.  **else**28.      add *B* to the selected solutions29.  **end if**
30. **end for**31.**End**

### 3.3. Fog Computing Closed Loop Model (FCCL)

This section presents our developed integrated objectives fog computing model FCCL. It is composed of five main sections. Section: 3.3.1 explains the first layer which is the fog interface. Section 3.3.2 is an overview of the task decomposer and task model. Next, Section 3.3.3 the task dispatcher. Then, Section 3.3.4 contains the network model, and lastly, Section 3.3.5 contains the optimization objectives.

**Figure 3:**
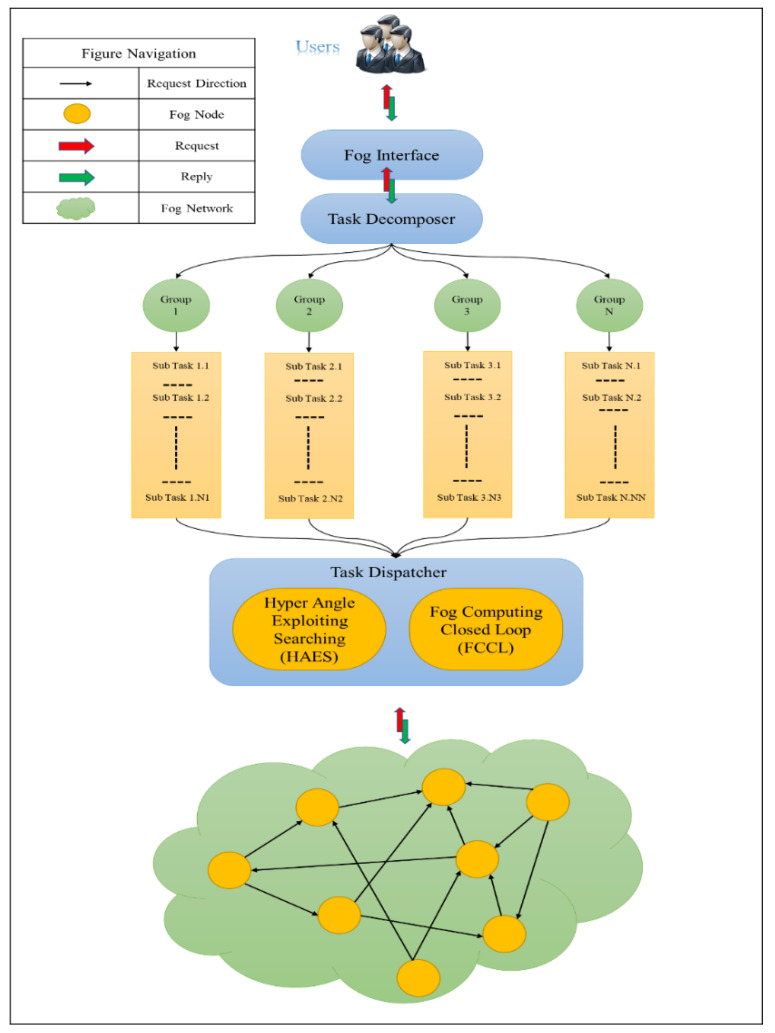
The framework of multi-criteria optimization for fog computing.

From a fog computing perspective, our problem is formulated similarly. The fog has an interface that receives from the user a request of executing a computational task with the needed criterion for optimization. Next, it calls an optimization algorithm that provides a set of non-dominated solutions with respect to the provided criteria. The user will make a decision for selecting one among them. The criteria are denoted by vectors $y=\left(y_{1},y_{2},\ldots y_{m}\right),$ where $\left\{y_{i}\right\}$ denotes a criterion for fog computing optimization. Without loss of generality, we consider five criteria, namely, Energy Consumption, Energy Distribution, Renting Cost, and Stability.

y=(energy consumption, energy distribution, renting cost, and stability). The solutions that are provided to the user gives the selected fog nodes for the execution of the request; we are represented by vector$\;x=\left(x_{1},x_{2},\ldots x_{n}\right)$. The goal is to maximize the domination aspect of the provided solutions and their diversity. This gives the user more variety of choices. To elaborate more, we present Figure 3, which elaborates the user giving a request to the user interface and waiting for a set of non-dominated solutions to select one. The fog interface communicates with the task decomposer that decomposes the task that is requested by the user to execute in the fog network. The role of the task decomposer is to partition the task into subsets of independent subtasks; we call each subset a group. Each group is executable independently on the other task.

This aspect enables shorter execution time, which is one of the metrics to be optimized. The task decomposer communicates with the task dispatcher that is responsible for calling the mathematical functions of the fog criterion for calculating the objective function for any candidate solution. Obviously, the task dispatcher receives the needed information from the fog network and the task decomposition and specification before carrying the optimization. The optimization is carried using a multi-objective optimization algorithm named HAES.

#### 3.3.1. Fog Interface

The fog interface will accept from the user two inputs. The first one is the task, and the second one is the preference vector of the various objectives for optimizing the task. The vector of preference between the five objectives is the five components vector, given as $pre=\left[pr_{1}\;pr_{2}\;pr_{3}\;pr_{4}\;pr_{5}\right]$ with the constraint$\;\sum_{i=1}^{5}pr_{i}=1$. The second input is the configuration input, which is also given by a vector named conf=[itMax popSize], where itMax denotes the maximum number of iterations, and popSize denotes the size of the population. Assuming that there is more interest in the time execution (makespan) and stability, the second interest is in the cost, and the third interest in the energy consumption and the energy balance, then the value of $pre=\left(1\times pr,1\times pr,\frac{1}{2}\times \mathrm{pr},\frac{1}{3}\times \mathrm{pr},\frac{1}{3}\times \mathrm{pr}\right)$. This implies, $1\times pr+1\times pr+\frac{1}{2}\times \mathrm{pr}+\frac{1}{3}\times \mathrm{pr}+\frac{1}{3}\times \mathrm{pr}=1.$ Then, pr=6/19.

#### 3.3.2. Task Decomposer and Task Model

**Figure 4:**
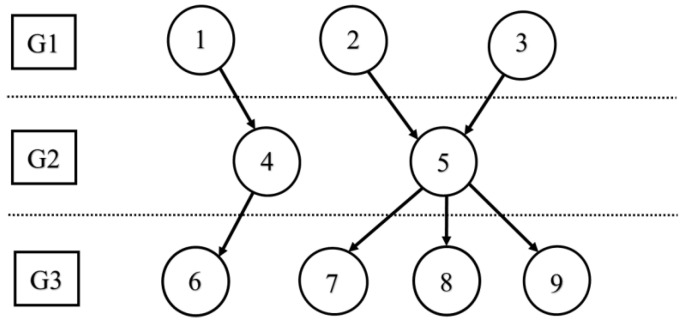
Task Decomposer.

The logical decomposition of data fusion tasks is a fundamental process in the design of systems aiming at combining multiple and heterogeneous cues collected by sensors. In recent years, a relevant body of research has focused on formalizing logical models for multi-sensor data fusion in order to propose appropriate and general task decomposition. Therefore, we suggest a task decomposer, which is elaborated in Figure 4, to decompose the data and classify based on priority. The role of the task’s decomposer is to decompose the tasks into a set of independent tasks; we denote them into groups $G=\left\{G_{1},G_{2},\ldots G_{N}\right\}$. Example 1 went particularly into decomposing and classifying the tasks.

This component has the role of accepting the task from the user. The task itself is modeled as a directed graph DG(V,E), where $V_{t}=\left\{t_{1},t_{2},\ldots t_{m}\right\}$, $E=\left\{e_{1},e_{2},\ldots e_{k}\right\},$ where m denotes the number of tasks in the graph and k denotes the number of directed edges. Where each edge $e_{i}=\left(t_{m1},t_{m2}\right),$ it denotes that $t_{m2}$ is dependent on $t_{m1}$. Another piece of information that is related to the task and has to be provided by the interface is the workload of tasks in terms of both computation and communication, where the computation is described by set $P=\left\{P_{1},P_{2},\ldots P_{m}\right\},$ where each $P_{i}$ denotes the computation that is the number of a clock for the task $P_{i},$ and the communication load is described by the set $L=\left\{L_{1},L_{2}\ldots .L_{m}\right\},$ where $L_{i}\;$represents the communication loads, which describes the total length of data to be exchanged among selected nodes for executing the task.

Example 1:

Task decomposer will classify the nodes in the network into groups, and each group depends on the number of nodes in the fog network. In addition, its direct graph, which is the fog nodes, will forward the request to the next node. The result of the task decomposer is set of three groups as $G_{1}$ = {1,2,3}, $G_{2\;}$ = {4,5}, $\mathrm{and}\;G_{3}$ = {6,7,8,9}. As we see, the tasks in each group are independent of each other, and they can be processed in any order.

#### 3.3.3. Task Dispatcher

The task dispatcher is responsible for allocating certain nodes in the fog network for the execution of the sub-tasks that result from the task decomposer. It contains the optimization algorithm HAES, which was presented in Section 3.2.3. The fog computing closed loop is presented in Section 3.3.

#### 3.3.4. Network Model

We assume that the network is an undirected graph $UDG\left(V_{n},E_{n}\right),$ where $V=\left\{n_{1},\;n_{2}\ldots n_{n}\right\},$ where n denotes the number of nodes in the network. $E=\left\{\left(n_{i},n_{j}\right)\right\}$ where $n_{i},n_{j}\in V$. Assuming that the nodes have wireless connections between each other, then we are interested in the distance between every two nodes. Each node i has a rate of computational energy consumption $e_{i}\;$[$\frac{j}{sec}$], and each two nodes $n_{i},n_{j}\in V$, have distance between them, which is given as $d_{ij}=d\left(n_{i},n_{j}\right).$ In addition, we assume that each node $n_{i}$ has a speed for execution $v_{i}$. Furthermore, we assume that each node has a maximum capacity for executing computational load $p_{0}$ and maximum capacity for executing communication load $l_{0}$.

#### 3.3.5. Optimization Objectives

We present in this section the equations of the optimization objectives. Our model has the aspect of integrating five objectives at the same time, which makes it distinguished from other models in the literature.

A. Time Latency

Time latency is an expression of how much time it takes for a packet of data to get from one designated point to another. It is sometimes measured as the time required for a packet to be returned to its sender, which is calculated by the following formula.

(5) $$T\;=\;\overset{m}{\underset{i=1}{\sum }}\overset{n}{\underset{j=1}{\sum }}t_{ij}\;\;\;$$

(6) $$t_{ij}\;=\;t_{ij}{}^{1}+t_{ij}{}^{2}$$

(7) $$t_{ij}{}^{1}\;=\;\frac{P_{ij}}{v_{i}}\;\mathrm{computation}\;\mathrm{time}$$

(8) $$t_{ij}{}^{2}\;=\;\frac{l_{ij}}{B}+t_{ij}{}^{queue}\;\mathrm{communication}\;\mathrm{time}$$

where $t_{ij}{}^{queue}$ denotes the queue waiting time. $P_{ij}$ denotes the task computational load that is assigned to node i. The speed is $v_{i}\;$of the node i. $l_{ij}$ denotes the communication load between i and j. Lastly, B denotes the bandwidth.

B. Energy Consumption

In order to send number packets from node A until node B, where the distance between the two nodes is d(A,B) = d, we calculate the consumed energy as Equation (9).

(9) $$e\left(A,B\right)\;=\;e\left(d\right)\;=\;\{\begin{array}{c} \left(e_{elec}+\varepsilon_{amp}d^{2}\right)l\left(A,B\right)\;for\;transmit\\ e_{elec}l\left(A,B\right)\;for\;receive\; \end{array}$$

where $e_{elec}$ denotes energy consumption for operating the radio model for each bit in the data. d denotes distance between the two nodes A,B. The coefficient of transmit amplifier given by $\varepsilon_{amp}$. l(A,B) denotes the number of bits to be sent from node A to node B.

Based on the term $e\left(A,B\right)\;=\;e_{A,B}$,$\;l\left(A,B\right)\;=\;l_{A,B}$, we can calculate the total energy consumption based on terms $E_{comp}$, $E_{comm},$ which represent the computation energy consumption and communication energy, respectively. The total energy is given in Equation (10), the computation energy is given in Equation (11), and the communication energy is given in Equation (12).

(10) $$E\;=\;E_{comp}+E_{comm}$$

(11) $$E_{comp}\;=\;\overset{n}{\underset{i\;=\;1}{\sum }}e_{i}t_{i}$$

where $e_{i}$ denotes to the energy consumption because of execution in node i, $t_{i}$ denotes to the time allocation of the node i, $e_{i,j}$ denotes the energy consumption because of communication between nodes i and j, and l(i,j) number of bits transferred between nodes i and j.

(12) $$E_{comm}=\overset{m}{\underset{i,j,\;i\neq j}{\sum }}e_{i,j}l_{i,j}$$

C. Energy Distribution

This term indicates the differences among the nodes in terms of the energy levels. The term is calculated as the standard deviation of the node’s energy as it is given in Equation (13).

(13) $$E_{\sigma \;}=\sqrt{\frac{\sum_{i=1}^{n}{\left(E_{i}-\bar{E}\right)}^{2}}{n-1}}$$

where $E_{i}$ denotes the consumed energy of node $i;\;\bar{E}$ denotes the average consumed energy of all nodes. n denotes the total number of nodes.

D. Renting Cost

The renting cost is defined as the total cost of rent, which is the summation of node i rental rate $r_{i}$ multiplied by the time of allocating the node according to Equation (14).

(14) $$C=\overset{n}{\underset{i=1}{\sum }}t_{i}r_{i}$$

where $r_{i}$ denotes the renting rate of the node$\;i.\;t_{i}$ denotes the time of allocating the node i.

E. Stability

This term indicates the total stability of the task execution. It is calculated as the summation of the reliability percentage of a certain node $rr_{i}$ multiplied by the time of allocating the nodes. The calculation is depicted in Equation (15).

(15) $$S=\overset{n}{\underset{i=1}{\sum }}t_{i}rr_{i}$$

where $rr_{i}$ denotes to the reliability rate of the node$\;n_{i}$, and $t_{i}$ denotes the time of allocating the fog node i.

F. Constraints

Before assigning any given solution to the fog network, it is needed to assure that it meets the constraints. Basically, there are two types of constraints that should be satisfied. The first one is the connectivity constraint, which states that any sub-network is assigned an execution of a task; it should be connected in order to execute the task that is assigned to the sub-network. The second constraint is named the load constraint. It states that for a task T with computational load P and communication load L, it should be allocated at least $N_{0}$ for execution. The value $N_{0}$ is calculated based on Equation (16).

(16) $$\{\begin{array}{c} N_{0}=Max\;\left(\frac{L}{L_{0}},\;\frac{P}{P_{0}}\right)\\ N\;\geq \;N_{0} \end{array}$$

## 4. Experimental Design and Parameters Setup

This section comprises three categories for presenting the evaluation of the proposed model and base benchmarks used in the evaluation. The first category, in Section 4.1, is the evaluation metrics of HAES and FCCL models. This section talks specifically about the most common and standard evaluation measures, which are hyper-volume, non-dominated solution, generational distance measure, inverse relative generational distance measure, delta metric measure, and set coverage measure. In addition, the parameters for HAES mode with base models. The second category, Section 4.2, is a dedicated section that presents the multi-objective mathematical functions that will test HAES and compare it with state-of-the-art approaches. The third, Section 4.3, presents the parameters for the FCCL model.

### 4.1. Evaluation Metrics of HAES and FCCL

This section presents the evaluation metrics that are used for evaluating our developed approaches, which are HAES and FCCL. Fog computing evaluation metrics are the same objectives that are used for optimization. We present the hyper-volume in sub-section A Next, we present the number of non-dominated solutions in sub-section B. Afterward, the generational distance is presented in sub-section C. Next, the inverse relative generational distance measure in sub-section D, and the delta metric is provided in sub-section E. Lastly, set coverage is giving in sub-section F.

A. Hyper Volume (HV) Measure

The hyper volume (HV) metric is widely used in evolutionary MOO to evaluate the performance of the searching algorithm [36]. It computes the volume of the dominated portion of the objective space related to the worst solution. This region is the union of the hypercube, with its diagonal as the distance between the reference point and a solution X from the Pareto Set (PS). High values of this measure present the desirable solutions. HV is presented by the following (Equation (17)):

(17) $$HV=\mathrm{volume}\left(\cup_{x\in P_{s}}Hyper\;Cube\;\left(x\right)\right).$$

B. Number of Non-Dominated Solutions (NDS)

The number of non-dominated solutions (NDS), which expresses the effectiveness of the optimization algorithm [37], can be calculated as the cardinality of PS as (18):

(18) $$NDS\left(N\right)=\left|P_{s}\right|.$$

C. Generational Distance Measure (GDM)

This metric, also called the GD metric [38], is a measure to evaluate the performance of a found Pareto Set (PS) compared with a reference point set (a true Pareto set (PS)). This measure is based on the distance among obtained solutions and reference points, which is calculated as follows (Equation (19)):

(19) GD(Ps,PT)= (∑i=1|Ps|di2)12|Ps|.

D. Inverse Relative Generational Distance Measure (IRGD)

Inverse Relative Generational Distance Measure (IRGD)

Another metric that is used is the inverse Relative Generational Distance or IRGD, and it is given in Equation (20).

(20) IRGD(Ps,PT)= |Ps|(∑i=1|Ps|di2)12.

E. Delta Metric Measure

The delta or diversity metric ∆ shows the extent to which it achieves the spread [14]. The delta measure receives the non-dominated set of solutions and provides the diversity metric, and can be computed according to the following equation:

(21) $$\Delta =\frac{d_{f}+d_{I}+\sum_{i=1}^{N-1}\left|d_{i}-d^{-}\right|}{d_{f}+d_{I}\left(N-1\right)d^{-}}$$

where N is the number of solutions, $d_{f}\;$and $d_{i},$ the Euclidean detachments between the extreme and border solutions, and d is all the consecutive distances, $d_{i}\;(i\;$= 1, 2,. . . , N −1). This measure is required to be slight and maintained to be less, because this measure indicates uniform distribution. In addition, it provides various selections to the decision-maker.

F. Set Coverage Measure

Set coverage measure [37], also called C metric, compares the Pareto sets *Ps1* and *Ps2* and can be identified by (22):

(22) $$C{\left(P_{s1},P_{s2}\right)}^{\;}=\frac{\left|\{y\in P_{s2}|Ǝx\in P_{s1}:y≺x\}\right|}{P_{s2}}$$

C equals the ratio of nondominated solutions in $P_{s2}$ dominated by non-dominated solutions in $P_{s1}$ to the number of solutions in$\;P_{s2}$. Thus, when evaluating a set Ps, the value of C(X; Ps) must be minimized for all Pareto sets X.

### 4.2. Multi-Objective Mathematical Functions

The algorithms are evaluated based on various relevant MOO mathematical functions. The formulas, optimization range, and true PF of each mathematical function are provided in Table 3. They have been used in most of the existing studies on MOO optimization as the benchmarking functions. The convexity is different for each function. Table 3 shows the bounds of the variables and the optimal solutions or PFs. In this way, our proposed approach can be validated against critical MMO measures. We selected three approaches, NSGA-II, NSGA-III, and MOGA-AQCD, which were presented in the background section, as the three relevant benchmarks to evaluate HAES.

To make the study quantitative, ten experiments are performed for each function using different seeds. This study also refers to the previous studies so that the same methodology of evaluation as of Multi-Objective Evolutionary Algorithms (MOEAs) is performed. The test function is chosen based on the well-known studies, including Fleming’s study (FON) [39], Kursawe’s study (KUR) [40], Poloni’s study (POL) [41], and Schaffer’s study (SCH) [42]. We then followed those guidelines and suggested six test problems, in which five of them are presented in Table 3, call ZDT1, ZDT2, ZDT3, ZDT4, and ZDT6. All problems have two objective functions, and none of these problems has any constraint. In addition, the number of variables, the bounds, the Pareto-optimal solutions, and the nature of the Pareto-optimal front for each problem.

The implementation is conducted using MATLAB 2019b. The parameters for NSGA-II, NSGA-III, MOGA-AQCD, and HAES are given in Table 4 and Table 5. The same number of solutions and generation was used for all the algorithms in order to have a fair comparison. An increase in the number of solutions and generations typically yields better performance results. The numbers of the population and generations are selected to be (100) and (500), respectively. The parameters of the crossover are determined based on two parts: fraction and ratio. The fraction is selected to be 2/n, where n denotes the solution length and the ratio is selected to be 1,2. For the scale of the mutation, we selected the value of 0,1. These values are the default ones that are used by the MATLAB optimization package.

These selected numbers are to obtain the PF within a balanced time. However, increasing both or one of them yields highly dominated solutions, given extensive exploration will be conducted in the searching space.

### 4.3. HAES Evaluation Based on FCCL Model

The evaluation is done based on population size 200 and number of generations 200. We run the model on 10 experiments. Each experiment is conducted on a different value of the quantization, α = {20, 23, 25, 28, 30, 33, 35, 37, 40, 45}. In addition, each experiment is repeated 10 times with different values of seed, which are given in Table 6. The results are decomposed into two sub-sections. The first one is the presentation of the results of the multi-objective mathematical functions, and the second one is the results of the evaluation of the fog computing closed-loop model.

## 5. Evolution and Enhanced Model Results

This part presents the results of the two models, HAES and FCCL, and discuss the experiment results comparing to the other models and their differences. Section 5.1 elaborates on the first phase which is the optimization of HAES with three benchmarks as follows NSGA-II, NSGA-III, and MOGA-AQCD; Section 5.2, the second phase, is the model of FCCL and the comparison of our model with the same benchmarks for phase one.

### 5.1. HAES Experimental Investigation and Results

**Figure 5:**
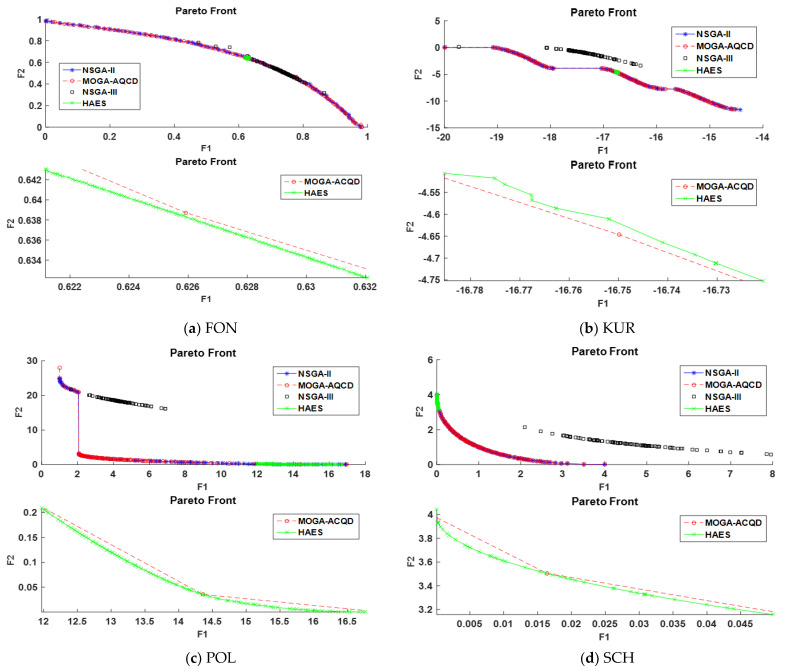

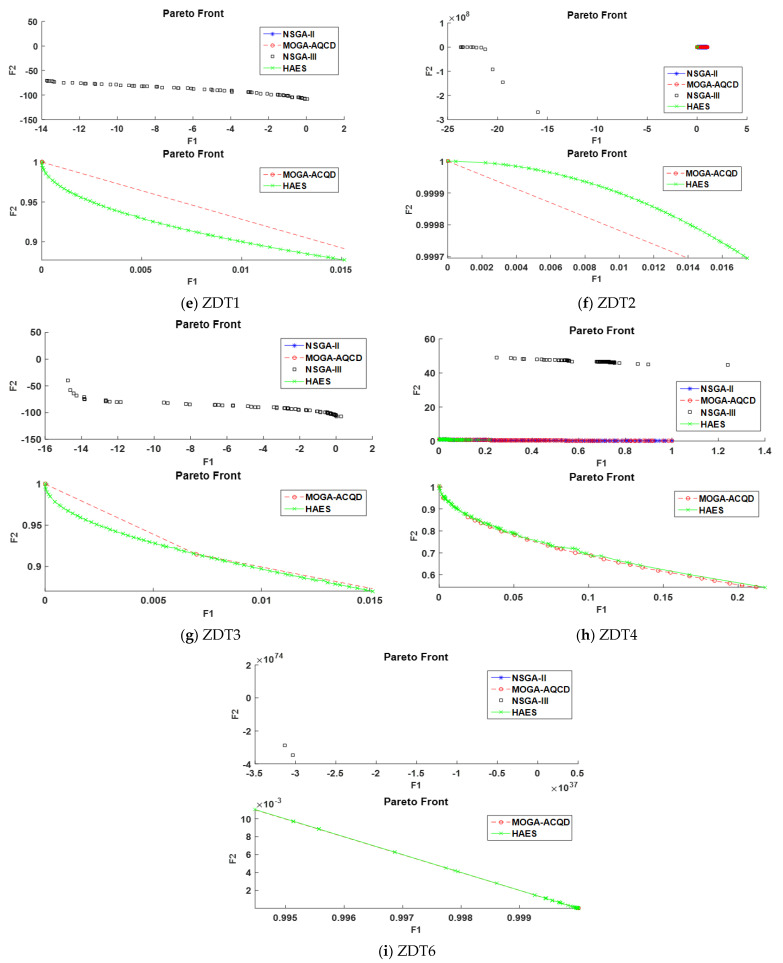
Pareto front with two scales: sub-figure HAES with MOGA-AQCD for FON, KUR, POL, SCH, ZDT1, ZDT2, ZDT3, ZDT4, and ZDT6.

The evaluation of the HAES algorithm is performed firstly based on mathematical functions with a challenging MOO nature as follows: FON, KUR, POL, SCH, ZDT1, ZDT2, ZDT3, ZDT4, and ZDT6. It presents the Pareto front, average hyper volume metric, average non-dominated solutions metric, an average of delta metric, and the average of generational distance metric, respectively, in each figure for HAES and other three benchmarks. As we observe in Figure 5, the Pareto front is plotted with two axes figures, because each of the mathematical functions has two objectives. Considering that HAES has an exploiting nature that enables the algorithm to each more dominant solution even if the regions of exploration were less, this has made it more capable of minimizing the values of the objectives.

In order to present this clearly, we show for each mathematical function two scales: the first one shows the general Pareto at the top and the second one shows the area of solutions found by HAES at the bottom. The Pareto front was lower for the functions FON, POL, SCH, ZDT1, ZDT2, ZDT3, and ZDT4, which is more domination with respect to these functions. The only function that has not achieved lower values of the Pareto front is KUR. However, HAES has achieved a more diverse Pareto front for KUR compared with the benchmarks. Figure 5 elaborate on the results for mathematical functions for each metric particularly.

In order to identify the superiority in terms of domination, we provide two tables: the first one is showing the domination of the benchmarks over HAES in Table 7, and the second shows the domination of HAES over the benchmarks in Table 8. As can be seen, the values in Table 7 are higher than their corresponding values in Table 8, which means that HAES is more dominant over the MOGA-AQCD, NSGA-III, and NSGA-II.

In order to assess the performance of HEAS in terms of the richness of the found solutions compared with the benchmarks, we present the hyper-volume. As it is shown in Table 9, ZDT6 has accomplished high hyper-volume only for KUR and ZDT6, while it was less for the other functions. This is interpreted as more domination of solutions that was accomplished for HAES compared with the benchmarks. This makes it more challenging to obtain high hyper-volume compared with MOGA-AQCD, NSGA-II, and NSGA-III, which has generated a lower dominant Pareto front.

In addition to hyper-volume, we generated an NDS measure that indicates the number of found solutions in the Pareto front. A higher value of NDS is equivalent to better performance in general. However, it is important to read NDS as a secondary metric after domination. We observe that HAES has accomplished competing values of NDS to the benchmarks for FON, POL, ZDT1, ZDT3, ZDT4, and ZDT6. Hence, it is considered a good performing algorithm from the perspective of not only domination, but also NDS.

The delta metric shows how much the solutions were equally distributed on the resultant Pareto front. A lower value of the delta metric implies a more equal distribution of the found solutions on the Pareto front. Considering that HAES’s focus is to search in an exploiting way, it provides lower distributed solutions in the Pareto front, which makes its value higher compared with the benchmarks and in general closer in order to the value of delta metric of NSGA-III. On the other side, we observe that NSGA-II and MOGA-AQCD have lower values of delta metric.

Another metric that is used to evaluate the performance of MOO is GD, which is preferred to be lower. It shows that HAES has accomplished lower GD for FON, POL, SCH, ZDT1, ZDT2, ZDT4, and ZDT6. We also observe that NSGA-III has suffered from relatively higher values of GD compared with the other approaches. It is important to point out that GD is not always correlated with the percentage of domination due to the change of scales between one objective and the other.

### 5.2. FCCL Investigation and Results

**Figure 6:**
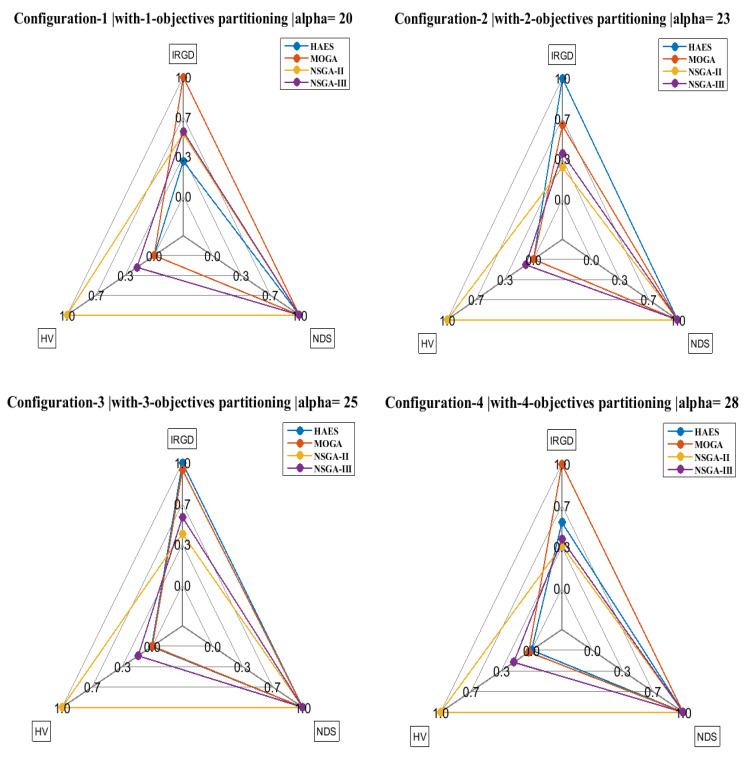

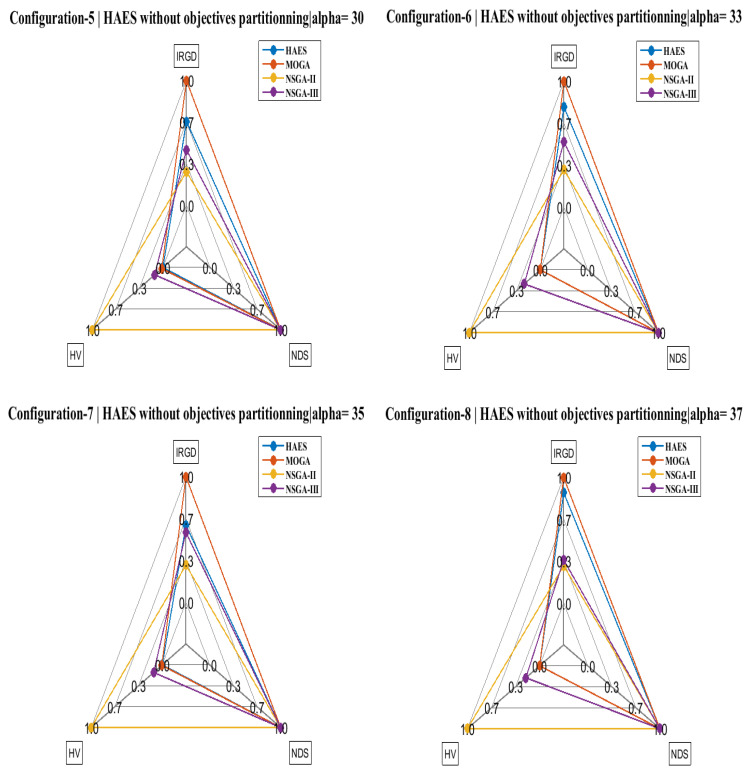

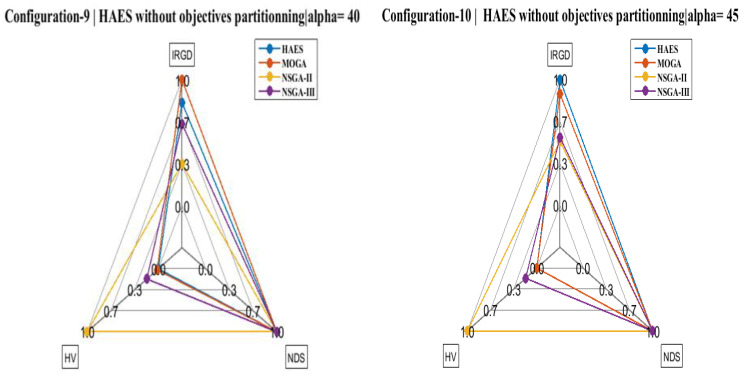
Comparison between HAES different configurations in terms of alpha and the other algorithms.

This section presents the evaluation of implementing HAES on the fog computing closed-loop model. Three main measures are presented for each of the provided configurations in the experimental design, namely, IRGD, which represented the inverse of the relative generational distance, HV, which represents the hyper volume, and NDS, which denotes the number of non-dominated solutions. The evaluation measures are presented with the different configurations in Figure 6. Looking at the figure, we observe that HAES was capable of accomplishing full IRGD and NDS for configurations 23, 25, 33, and 45. Additionally, we observe that HAES’ different configuration was not able to bring HV to its maximum value.

**Figure 7:**
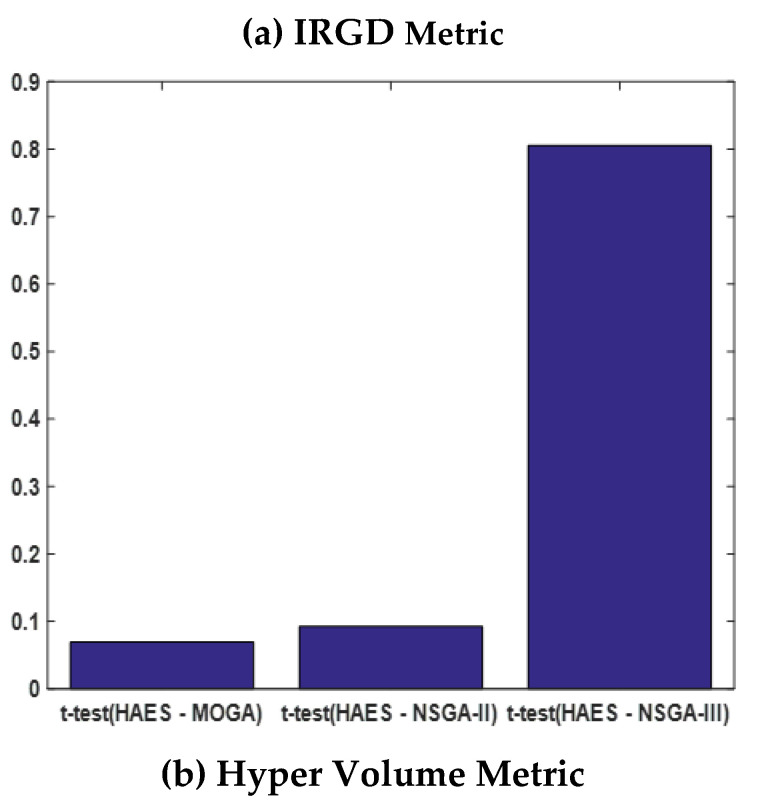

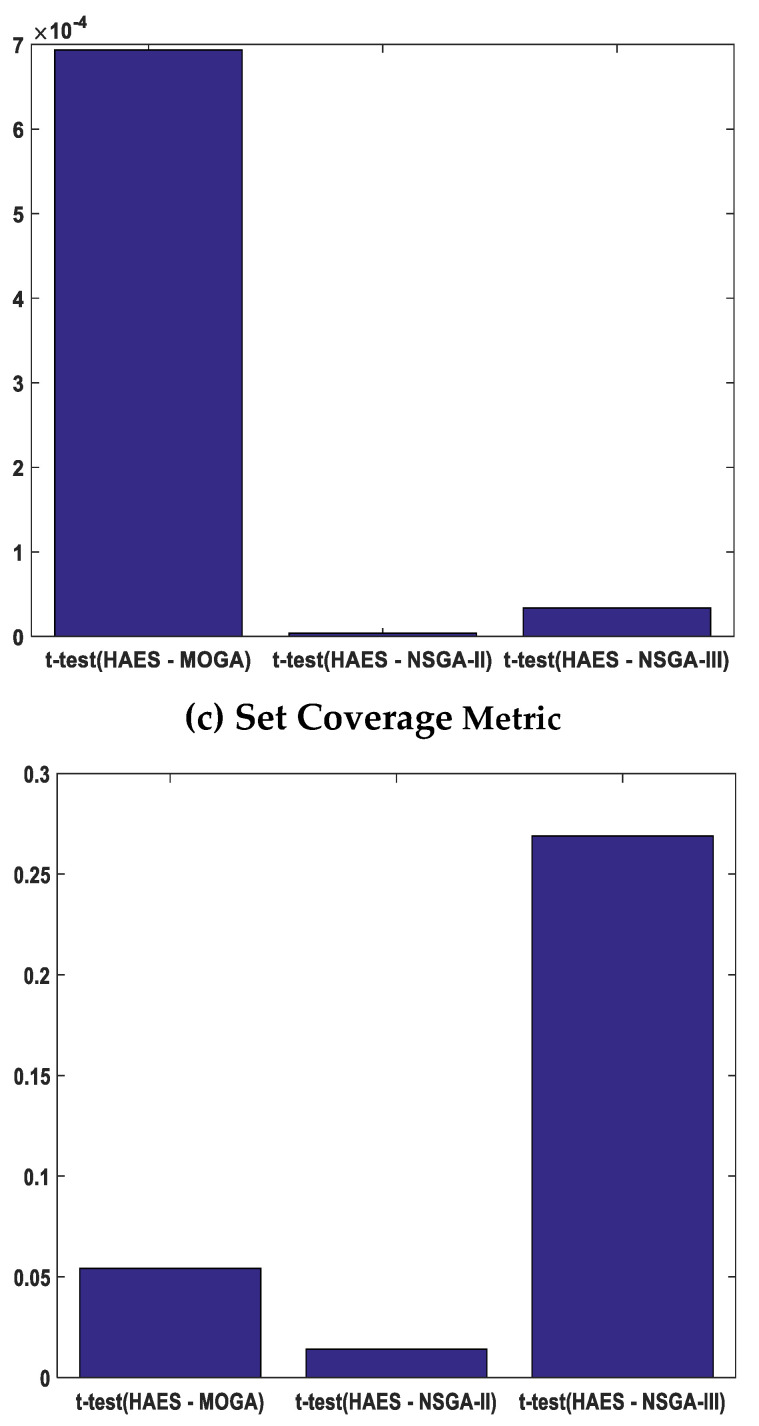
*t*-test to compare the performance of HAES and MOGA-AQCD, NSGA-II, and NSGA-III.

For a more quantitative comparison of the difference in the performance between HAES and other benchmarks, we generated the results of the t-test in Figure 7 for three metrics: IRGD, HV, and set coverage. Their values reveal that HAES has outperformed other benchmarks with respect to set coverage with a confidence of more than 70%, and with respect to IRGD with a confidence of more than 90%. However, HAES was less superior with respect to HV, with a confidence of more than 90%.

Looking at the hyper-volume as a secondary measure after the domination and considering that reaching more optimal solutions might limit their spread in the objective space, we interpret that hyper-volume of HAES has not outperformed the hyper-volume of the benchmarks. However, we could have accomplished more optimal solutions with HAES compared with the benchmarks, as both IRGD and set-coverage of HAES have outperformed their corresponding values in the benchmarks.

## 6. Conclusions and Future Works

This article has presented a novel formulation of the problem of fog computing optimization with a multi-objective perspective. The covered objectives are the time latency, the energy consumption with the energy distribution, the renting cost, and stability. The multi-objective and the conflicting nature of the problem require adopting meta-heuristic searching for solving it. However, due to the relatively high number of objectives, different from the relevant existing studies in literature, this research has proposed a novel hyper-angle genetic optimization. The role of the hyper angle is to prioritize solutions within the same rank based on their best-accomplishing rank, which gives the algorithm more exploitive capability. In addition, the article has adopted the concept of objective decomposition by evaluating the approach on various sizes of sub-set of objectives for the objective’s decomposition. Objective decomposition enables exploring the boundary of the objective space before going to the intermediate region while searching. Such an approach is crucial for the relatively large number of objectives. Furthermore, various values of angle resolutions were used for the evaluation. It was found that the number of sub-set of objectives while performing the objectives decomposition as well as the value of the angle play an important role in the overall performance. The approach is limited in its dependency on static parameters for both. Hence, our planned future work is to enable an adaptive number of objectives, in which the value of the angle is investigated.

## Figures and Tables

**Table 1:** Summary of the covered objectives in the fog computing model in the literature.

Authors/Objectives	Energy Consumption	Renting Cost	Stability	Time Latency	Energy Distribution
[32]	✘	✘	✓	✓	✘
[33]	✘	✘	✓	✘	✓
[34]	✓	✘	✘	✓	✘
[35]	✘	✓	✘	✓	✘
Proposed Model	✓	✓	✓	✓	✓

**Table 2:** Terms and symbols used for presenting the mathematical models.

Symbol	Meaning
$DG\left(V_{t},E_{t}\right)$	Graph of tasks.
$V=\left\{t_{1},t_{2},\ldots t_{m}\right\}$	Tasks to be executed in the fog network.
$E=\left\{e_{1},e_{2},\ldots e_{k}\right\}$	The dependency relation between the tasks.
$e_{i}=\left(t_{m1},t_{m2}\right)$	A connection between task $t_{m1}$ and $t_{m2}$.
$P=\left\{P_{1},P_{2},\ldots P_{m}\right\}$	Computation load of the task.
$L=\left\{L_{1},L_{2}\ldots .L_{m}\right\}$	Communication loads of the task.
$G=\left\{G_{1},G_{2},\ldots G_{n}\right\}$	Subsets of independent graphs of tasks (a task in any graph can be executed with any order comparing with other tasks in the same graph).
$V=\left\{v_{1},v_{2},\ldots v_{n}\right\}$	Speed of CPU of nodes in the network.
$UDG\left(V_{n},E_{n}\right)$	Graph of nodes.
$V=\left\{n_{1},\;n_{2}\ldots n_{n}\right\}$	Nodes are available for service in the fog network.
$RC=\left\{r_{1},\;r_{2}\ldots r_{n}\right\}$	Renting cost of nodes.
$RR=\left\{rr_{1},\;rr_{2}\ldots rr_{n}\right\}$	Reliability of nodes.
$E_{comp}$	Energy consumption because of the computational load.
$E_{comm}$	Energy consumption because of communication.
B	The bandwidth of the connection’s links between nodes that participate in executing the task.
$E_{\sigma \;}$	Energy balance is represented by the standard deviation of the energy
C	The cost, which is represented by the total rental cost.
S	The stability term is a measure of the reliability of the nodes that execute the task.
$d=\left[d_{ij}\right]=[d\left(n_{i},n_{j}\right)$]	The distance information between every two nodes
$e=\left[e_{i}\right]$	The energy consumption rate of nodes in the network
$P_{0}$	The maximum computational load that can be given to a certain node
$L_{0}$	The maximum communication load that can be given to a certain node

**Table 3:** Mathematical functions for evaluating MOO measures.

Problem	n	Variable Bounds	Objective Function	Optimal Solution	Remark
FON	3	[−4, 4]	$f1\;\left(x\right)=1-\exp\left(-\overset{3}{\underset{i=1}{\sum }}\;\;{\left(xi-\frac{1}{\frac{1}{\sqrt{3}}}\right)}^{2}\right)$ $f2\;\left(x\right)=1-\exp\left(-\overset{3}{\underset{i=1}{\sum }}\;\;{\left(xi-\frac{1}{\frac{1}{\sqrt{3}}}\right)}^{2}\right)$	$x_{1}=x_{2}=x_{3}$	Non-convex
KUR	3	[−5, 5]	$f_{1}\left(x\right)=\overset{n-1}{\underset{i=1}{\sum }}\left(-10\;exp\left(-0.2\;\sqrt{x_{i}^{2}+x_{i}^{2}+1}\right)\right)$ $f\left(x\right)=\overset{n}{\underset{i=1}{\sum }}\left(|x_{i}|{\;}^{0.8}+5\sin x_{i}^{3}\right)$	[43]	Non-convex
POL	2	[−π, π]	$f_{1}\left(x\right)=\left[1+{\left(A_{1}-B_{1}\right)}^{2}+{\left(A_{2}-B_{2}\right)}^{2}\right]$ $f_{2}\left(x\right)=\left[{\left(x_{1}+3\right)}^{2}+{\left(x_{2}+1\right)}^{2}\right]$	[43]	Non-convexDisconnected
SCH	1	[$10^{-3},\;\;10^{3}]$	$f_{1}\left(x\right)=x^{2}$ $f_{2}\left(x\right)={\left(x-2\right)}^{2}$	X ∈[0, 2]	Convex
ZDT1	30	[0, 1]	$f_{1}\left(x\right)=\overset{n-1}{\underset{i=1}{\sum }}(-10\;\exp(-0.2\;\sqrt{x_{i}^{2}+x_{i+1}^{2}}$ $f_{2}\;\left(x\right)=\overset{n}{\underset{i=1}{\sum }}(\;\left|x_{i}\right|{\;}^{0.8}+5\sin x_{i}^{3})$	$x_{1}\in \left[0,\;1\right]$ $x_{i}=0$ i=2, 3,….,n	Convex
ZDT2	30	[0, 1]	$f_{1}\left(x\right)=x_{1}$ $f_{2}\left(x\right)=g\left(x\right)\left[1-{\left(x_{1}/g_{x}\right)}^{2}\right]$ $g\left(x\right)=1+9\;\left(\overset{n}{\underset{i=2}{\sum }}x_{i}\right)/\left(n-1\right)$	$x_{1}\in \left[0,\;1\right]$ $x_{i}=0$ i=2, 3,….,n	Non-convex
ZDT3	30	[0, 1]	$f_{1}\left(x\right)=x_{1}$ $f_{2}\left(x\right)=g\left(x\right)\left[1-{\sqrt{\frac{x_{1}}{g\left(x\right)}\;\;\;}}^{\;}-\frac{x_{1}}{g\left(x\right)}\;\sin\left(10\;\pi \;x_{1}\right)\;\;\right]$ $g\left(x\right)=1+9\;\left(\overset{n}{\underset{i=2}{\sum }}x_{i}\right)/\left(n-1\right)$	$x_{1}\in \left[0,\;1\right]$ $x_{i}=0$ i=2, 3,….,n	Convex, Disconnected
ZDT4	10	[0, 1] [−5, 5]	$f_{1}=x_{1}$ $f_{2}=g\left(x\right)\left[1-{\left(\frac{f_{1}}{g}\right)}^{0.5}\right]$ $g=1+10\left(N-1\right)+\overset{N}{\underset{i=2}{\sum }}\left(x_{i}^{2}-10\cos\left(4\pi x_{i}\right)\right)$		Non-convex
ZDT6	10	[0, 1]	$f_{1}\left(x\right)=1-\exp\left(-4x_{1}\right)\sin^{6}\;\left(6\pi x_{1}\right)$ $f_{2}\left(x\right)=g\left(x\right)\left[1-{\left(\frac{f_{1}\left(x\right)}{g\left(x\right)}\right)}^{2}\right]$ $g\left(x\right)=1+9\;\left[\left(\overset{n}{\underset{i=2}{\sum }}x_{i}\right)/\left(n-1\right)\right]\begin{array}{c} {\;}^{0.25} \end{array}$	$x_{1}\in \left[0,1\right]$ $x_{i}=0$ i=2, 3,….,n	Convex, non-uniformly spaced

**Table 4:** Parameters of NSGA-II, MOGA-AQCD, and HAES.

Parameters		NSGA-II	MOGA-AQCD	HAES
No. of solution		100	100	100
No. of generation		500	500	500
Crossover option	Fraction	2/n	2/n	2/n
	Ratio	1,2	1,2	1,2
Mutation option	Fraction	2/n	2/n	2/n
	Scale	0,1	0,1	0,10
	Shrink	0.5	0.5	0.5
Quantification of angle space (α)		N/A	$10^{-7}$ for all test except KUR$\;5\times 10^{-7}$	$10^{-7}$ for all test except KUR$\;5\times 10^{-7}$

**Table 5:** Parameters of NSGA-III.

Parameters		NSGA-III
No. of Solution		100
No. of Generation		500
Crossover Percentage		0.5
Mutation Option	Mutation Percentage	0.5
	Mutation Rate	0.02
Number of Divisions		10

**Table 6:** Table of parameters used for evaluation FCCL Model.

Parameter	Value
Population size	200
Number of generations	200
Number of random experiments	10
α	{20, 23, 25, 28, 30, 33, 35, 37, 40, 45}
Number of nodes	30
Number of tasks	6
Number of objectives	5
Crossover	1.2
Mutation	0.5, 1.5

**Table 7:** Average set coverage values of HAES compared to those of MOGA-AQCD, NSGA-III, and NSGA-II.

Functions	MOGA-AQCD	NSGA-III	NSGA-II
FON	1.100 × 10^−2^	3.000 × 10^−2^	1.500 × 10^−2^
KUR	3.100 × 10^−2^	2.290 × 10^−1^	2.700 × 10^−2^
POL	8.000 × 10^−3^	1.000 × 10^−3^	4.000 × 10^−2^
SCH	2.000 × 10^−3^	6.880 × 10^−1^	2.000 × 10^−3^
ZDT1	0.000 × 10^−0^	0.000 × 10^−0^	1.500 × 10^−2^
ZDT2	0.000 × 10^−0^	0.000 × 10^−0^	0.000 × 10^−0^
ZDT3	6.000 × 10^−3^	0.000 × 10^−0^	1.500 × 10^−2^
ZDT4	4.815 × 10^−2^	6.000 × 10^−1^	9.000 × 10^−2^
ZDT6	2.750 × 10^−1^	0.000 × 10^−0^	2.710 × 10^−1^

**Table 8:** Average set coverage of MOGA-AQCD, NSGA-III, and NSGA-II compared to that of HAES.

Functions	MOGA-AQCD	NSGA-III	NSGA-II
FON	0.000 × 10^−0^	0.000 × 10^−0^	0.000 × 10^−0^
KUR	8.140 × 10^−3^	7.488 × 10^−3^	1.279 × 10^−2^
POL	1.000 × 10^−3^	0.000 × 10^−0^	1.300 × 10^−2^
SCH	2.000 × 10^−3^	0.000 × 10^−0^	2.000 × 10^−3^
ZDT1	0.000 × 10^−0^	1.000 × 10^−0^	3.000 × 10^−3^
ZDT2	0.000 × 10^−0^	1.000 × 10^−0^	0.000 × 10^−0^
ZDT3	0.000 × 10^−0^	1.000 × 10^−0^	0.000 × 10^−0^
ZDT4	0.0481209	0	0.1139833
ZDT6	0	1	0

**Table 9:** Average of MOO metrics for benchmarking mathematical functions.

Problems	Evaluation Measure	HAES	MOGA-AQCD	NSGA-III	NSGA-II
FON	Average of Hyper Volume	**5.685**	0.298	0.089	0.297
	Average Non-Dominated Solutions	100	100	100	100
	Delta Metric	0.991	**0.196**	1.011	0.281
	Average Generational Distance	**0.00109**	0.001199	0.001483	0.001199
KUR	Average of Hyper Volume	15.85	25.66	2.316	**25.67**
	Average Non-Dominated Solutions	61.8	**100**	**100**	**100**
	Delta Metric	0.8695	**0.3695**	1.035	0.4129
	Average Generational Distance	0.01893	0.006606	0.07131	**0.006420**
POL	Average of Hyper Volume	0.4963	368.2	17.45	**369.1**
	Average Non-Dominated Solutions	100	100	100	100
	Delta Metric	**0.9289**	1.308	1.026	0.9444
	Average Generational Distance	**0.001193**	0.007846	0.204	0.008936
SCH	Average of Hyper Volume	0.02784	13.26	**17.45**	13.26
	Average Non-Dominated Solutions	100	100	100	100
	Delta Metric	1.057	**0.6812**	1.021	0.6812
	Average Generational Distance	0.001227	**0.0008915**	1.15	**0.0008915**
ZDT1	Average of Hyper Volume	0.0012	0.6591	**187.1**	0.6579
	Average Non-Dominated Solutions	**100**	**100**	66	**100**
	Delta Metric	0.9863	**0.4984**	0.9223	0.6562
	Average Generational Distance	7.92 × 10^−4^	**4.18** × 10^−4^	10.9096	5.02 × 10^−4^
ZDT2	Average of Hyper Volume	1.6993	0.3274	0.3247	**2.1159**
	Averages Non-Dominated Solutions	**100**	**100**	13.8	**100**
	Delta Metric	0.9985	**0.3258**	1.295	0.6794
	Average Generational Distance	0.0011	**5.06** × 10^−4^	2.31 × 10^11^	5.31 × 10^−4^
ZDT3	Average of Hyper Volume	0.0012	0.7763	341.5	0.7771
	Average Non-Dominated Solutions	**100**	**100**	39.1	**100**
	Delta Metric	0.9915	0.7661	0.9718	**0.7541**
	Average Generational Distance	**5.55** × 10^−4^	6.81 × 10^−4^	14.3872	6.60 × 10^−4^
ZDT4	Average of Hyper Volume	0.2211	0.6407	**0.829**	0.6119
	Average Non-Dominated Solutions	87.3	**100**	67.6	**100**
	Delta Metric	1.014	0.4384	1.013	**0.3854**
	Average Generational Distance	**9.05** × 10^−4^	0.0012	7.9171	09.05 × 10^−4^
ZDT6	Average of Hyper Volume	**0.4746**	0.2646	0	0.2636
	Average Non-Dominated Solutions	59.8	**100**	1.4	**100**
	Delta Metric	1.214	**0.635**	0.9666	**0.7989**
	Average Generational Distance	0.0363	3.35 × 10^−4^	3.47 × 10^85^	**3.20** × 10^−4^

## Data Availability

The data that support the findings of this study are available from the corresponding author, [RH], upon reasonable request.
